# THOC5, a member of the mRNA export complex, contributes to processing of a subset of wingless/integrated (Wnt) target mRNAs and integrity of the gut epithelial barrier

**DOI:** 10.1186/1471-2121-14-51

**Published:** 2013-11-22

**Authors:** Shashank Saran, Doan DH Tran, Sabine Klebba-Färber, Patricia Moran-Losada, Lutz Wiehlmann, Alexandra Koch, Himpriya Chopra, Oliver Pabst, Andrea Hoffmann, Robert Klopfleisch, Teruko Tamura

**Affiliations:** 1 Institut fuer Biochemie, Medizinische Hochschule Hannover, OE4310 Carl-Neuberg-Str. 1, Hannover D-30623, Germany; 2 Pädiatrische Pneumologie, Medizinische Hochschule Hannover, OE6710 Carl-Neuberg-Str. 1, Hannover D-30623, Germany; 3 Institut fuer Immunologie, Medizinische Hochschule Hannover, OE5240 Carl-Neuberg-Str. 1, Hannover D-30623, Germany; 4 Unfallchirurgie, Medizinische Hochschule Hannover, Carl-Neuberg-Str. 1, Hannover D-30623, Germany; 5 Institute of Veterinary Pathology, Freie Universitaet Berlin, Robert-von-Ostertag- Str. 15, Berlin D-14163, Germany

**Keywords:** mRNA export protein THOC5, Tamoxifen inducible knockout mice, Gastrointestinal tract, Wnt target mRNAs, Sepsis

## Abstract

**Background:**

THO (Suppressors of the transcriptional defects of hpr1 delta by overexpression) complex 5 (THOC5), an mRNA export protein, is involved in the expression of only 1% of all genes. Using an interferon inducible knockout mouse system, we have previously shown that THOC5 is an essential element in the maintenance of hematopoietic stem cells and cytokine-mediated hematopoiesis in adult mice. Here we interrogate THOC5 function in cell differentiation beyond the hematopoietic system and study pathological changes caused by THOC5 deficiency.

**Results:**

To examine whether THOC5 plays a role in general differentiation processes, we generated tamoxifen inducible THOC5 knockout mice. We show here that the depletion of THOC5 impaired not only hematopoietic differentiation, but also differentiation and self renewal of the gut epithelium. Depletion of the THOC5 gene did not cause pathological alterations in liver or kidney.

We further show that THOC5 is indispensable for processing of mRNAs induced by Wnt (wingless/integrated) signaling which play key roles in epithelial cell differentiation/proliferation. A subset of Wnt target mRNAs, SRY-box containing gene 9 (*Sox9),* and achaete-scute complex homolog 2 (*Ascl2),* but not Fibronectin 1 *(Fn1),* were down-regulated in THOC5 knockout intestinal cells. The down-regulated Wnt target mRNAs were able to bind to THOC5. Furthermore, pathological alterations in the gastrointestinal tract induced translocation of intestinal bacteria and caused sepsis in mice. The bacteria translocation may cause Toll-like receptor activation. We identified one of the Toll-like receptor inducible genes, prostaglandin-endoperoxidase synthase 2 (*Ptgs2* or *COX2*) transcript as THOC5 target mRNA.

**Conclusion:**

THOC5 is indispensable for processing of only a subset of mRNAs, but plays a key role in processing of mRNAs inducible by Wnt signals. Furthermore, THOC5 is dispensable for general mRNA export in terminally differentiated organs, indicating that multiple mRNA export pathways exist. These data imply that THOC5 may be a useful tool for studying intestinal stem cells, for modifying the differentiation processes and for cancer therapy.

## Background

The THO complex, which is a sub-member of TREX (transcription/export), was originally identified in *Saccharomyces cerevisiae* as a five protein complex (Tho2p, Hpr1p, Mft1p, Thp2p, and Tex1) [1-6] that plays a role in transcriptional elongation, nuclear RNA export and genome stability. In higher eukaryotes such as *Drosophila melanogaster*[7] or humans [8], three proteins, (THOC1/hHpr1/p84, THOC2/hRlr1, and THOC3) and three additional unique proteins were identified, namely THOC5/Fms interacting protein (FMIP) [9], THOC6 and THOC7, as members of the THO complex. However, it is still unclear whether all members of THO complex play a role as one functional unit.

We have previously shown that THOC5 is a substrate for several tyrosine kinases such as macrophage-colony stimulating factor (M-CSF, or CSF-1) receptor, Fms [9], and various leukemogenic tyrosine kinases, such as Bcr-Abl (breakpoint cluster region-Abl tyrosine kinase fusion protein), translocation-ets-leukemia (TEL)-platelet derived growth factor (PDGF) receptor, or nucleophosmin (NPM)-anaplastic lymphoma kinase (ALK) [10,11]. In addition, we have recently shown that DNA damage causes loss of the RNA binding potential of THOC5 [12], and protein kinase C phosphorylates and inhibits nuclear import of THOC5 [13], suggesting that THOC5 is regulated by extracellular signalling. Furthermore, the ectopic expression or the depletion of THOC5 in mouse myeloid progenitor or mesenchymal progenitor cell lines causes abnormal hematopoiesis or abnormal muscle differentiation, respectively, suggesting that the expression level of THOC5 is important for the normal differentiation process [9,10,14,15]. We have previously identified THOC5 dependent mRNAs in the fibroblast system [16]. Surprisingly, only 71 genes were downregulated by depletion of THOC5. However, over 40% of these genes were involved in differentiation processes. Furthermore, we recently examined THOC5 dependent mRNAs in monocytes/macrophages. In this system also, only 99 genes were down-regulated upon depletion of THOC5 [17]. Along the same line, depletion of THOC5 does not affect bulk poly (A) + RNA export [18] and it has been recently shown that the knockdown of THOC5 in Hela cells leads to down-regulation of 289 genes [19].

Using interferon inducible THOC5 knockout mice, we have previously shown that the depletion of the THOC5 gene causes rapid apoptosis of hematopoietic cells, but not of any other organs. After bone marrow transplantation, mice survived for more than 3 months without any symptoms, suggesting that THOC5 plays a key role in maintaining hematopoietic cells. However, since interferon is one of the important cytokines for hematopoiesis [20], the observed phenotype may be due to the synergistic effects of interferon and knockdown of THOC5. Furthermore, depletion of the THOC5 gene resulting from treatment with poly I:C is limited to certain organs.

We therefore generated tamoxifen inducible THOC5 knockout mice. The treatment of mice with tamoxifen caused deletion of THOC5 exons 4/5 from bone marrow, colon, stomach, jejunum, liver, and kidney. We show here that THOC5 not only plays a key role in hematopoiesis, but also in another regenerative organ, the gastrointestinal tract. On the other hand, depletion of THOC5 in terminally differentiated organs such as liver or kidney did not result in any pathological alterations, nor did it influence mRNA export. Finally, we show that a subset of wingless/integrated (Wnt) target mRNAs is THOC5 dependent. These data suggest that mRNA export protein THOC5 controls the differentiation in a wide range of regenerative organs.

## Results and discussion

### THOC5 exons 4/5 were deleted from bone marrow, colon, liver, and kidney, but not from lung after tamoxifen treatment

**Figure 1:**
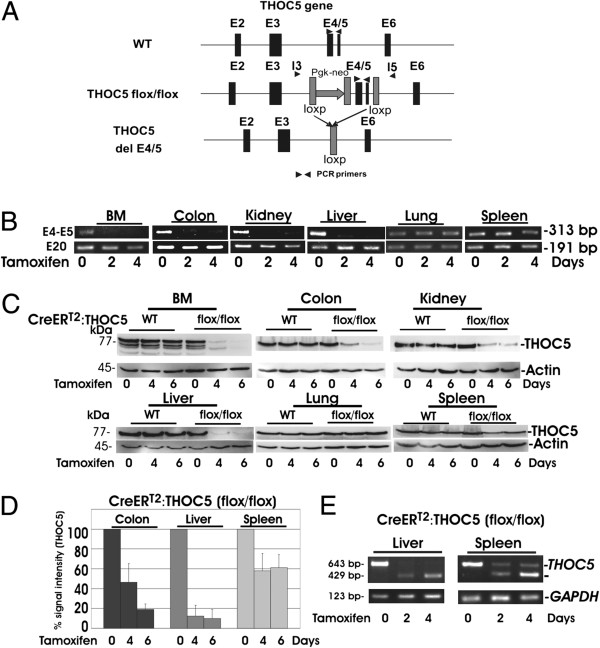
**THOC5 exons 4/5 were deleted from BM, colon, kidney, and liver after tamoxifen treatment. (A)** The genomic structure of the exon 2 (E2) to exon 6 (E6) THOC5 locus is depicted. THOC5 deficient mice were generated as described previously [23]. The loxP sites are located before E4 and after E5. Rosa26-*Cre*ER^T2^ (wild type (wt)) (n = 12) and Rosa26-*Cre*ER^T2^:THOC5 (flox/flox) (flox/flox) (n = 12) mice were injected with tamoxifen i. p. 2 times at 3-day intervals and were sacrificed 0, 2, 4 and 6 days after the first tamoxifen injection (3 mice for each preparation). Genomic DNA, protein and RNA were isolated from the same organs. **(B)** Genomic DNAs were supplied for the determination of deletion of exons 4/5 by PCR. PCR product: wild type allele: 313 bp; THOC5 E4/E5 del allele: 0 bp. As control, E20 specific primers (product: 191 bp) were supplied. **(C)** Protein was extracted from organs as indicated and subjected to THOC5 and actin specific immunoblot. Total protein amount was standardized by actin specific immunoblot. We performed 3 independent experiments and we show one example of representative data. **(D)** THOC5 and actin specific bands were quantified using the TINA 2.0 software (see Methods-Immunoblot Procedures). The signal intensity of THOC5 was standardized by actin band of each sample (3 mice for each preparation). The reduced percent signal intensity from mice treated with tamoxifen are shown. Mean values from three independent experiments are shown. The error bars represent standard deviation. **(E)** RNAs were extracted from liver or spleen of same mice as described above, and supplied for *THOC5 (*wt: 643 bp or delta E4/E5:429 bp) and *GAPDH* (123 bp) specific RT-PCR.

To extend previous work on THOC5 function to a broader range of organs and cell types, we generated a novel inducible THOC5 knockout mouse strain. We used tamoxifen-induced cre-recombinase expression in Rosa26-*Cre*ER^T2^ mice [21]. In Rosa26-*Cre*ER^T2^ mice, the *Cre*ER^T2^ fusion gene is under the control of the ROSA26 promoter and hence ubiquitously expressed in all organs. However, gene recombination is only induced by injection of the synthetic ligand, tamoxifen that leads to formation of an active cre-recombinase [21]. THOC5 (flox/flox) mice (Figure 1A) were mated to Rosa26-*Cre*ER^T2^ mice, generating tamoxifen inducible THOC5 knockout mice. The deletion mutation of THOC5 was induced by intraperitoneal (i.p.) injection with tamoxifen twice at 2-day intervals. We injected 5-9-week old Rosa26-*Cre*ER^T2^ (n = 12) and Rosa26-*Cre*ER^T2^:THOC5 (flox/flox) mice (n = 12) with tamoxifen. Two or four days after the first tamoxifen injection, mice were sacrificed and deletion of a functional THOC5 was analyzed by PCR using exons 4/5 specific primers in genomic DNAs from bone marrow (BM), colon, kidney, liver, lung and spleen. As control, exon 20 specific PCR was performed. In all control Rosa26-*Cre*ER^T2^ mice and Rosa26-*Cre*ER^T2^:THOC5 (flox/flox) mice without tamoxifen treatment, an exon 4/5 specific product (313 bp) and exon 20 specific product (191 bp) were detected from all organs. THOC5 exons 4/5 were deleted from bone marrow (BM), colon, kidney and liver of Rosa26-*Cre*ER^T2^:THOC5 (flox/flox) mice within 2 days after tamoxifen treatment, but not from lung and spleen (Figure 1B). To examine whether THOC5 exons 4/5 were only partially deleted in lung and spleen, genomic DNAs from lung and spleen were analyzed by PCR using introns 3/5 specific primers. Corresponding bands gave 1110 bp (with exons 4/5) and 355 bp sizes (lacking exons 4/5), respectively. A PCR product lacking exons 4/5 (355 bp) was detected in both organs within 2 days (data not shown), indicating that THOC5 exons 4/5 were only partially deleted in lung and spleen.

We next examined THOC5 at the protein level. The deletion of exons 4/5 of THOC5 causes a frame shift of product and the truncated protein is expected to be only 83 amino acids long and lacking the THOC1 binding domain [22]. However, we did not detect the truncated small THOC5 product in all organs. The level of THOC5 protein did not change in organs from Rosa26-*Cre*ER^T2^ control mice before or after tamoxifen treatment, however in Rosa26-*Cre*ER^T2^:THOC5 (flox/flox) bone marrow, colon, stomach, jejunum, kidney and liver the level of THOC5 was reduced to less than 20% within 6 days in all mice (Figure 1C and D; stomach and jejunum: data not shown). The level of THOC5 product in spleen was reduced by only 40% (Figure 1C and D). We then examined the THOC5 mRNAs in spleen. As control, we applied mRNA obtained from liver in which the THOC5 product was reduced to 10% of control liver within 4 days after tamoxifen treatment (Figure 1E). As expected, from liver only mRNA without exons 4/5 (429 bp) was detected after tamoxifen treatment. In agreement with data obtained from genomic DNA and protein (Figure 1B and C), both mRNA with (643 bp) and without (429 bp) exons 4/5 were detected in spleen 2 and 4 days after tamoxifen treatment, suggesting that THOC5 is deleted in a particular population of spleen cells. Notably, in interferon inducible THOC5 knockout mice, THOC5 was depleted in bone marrow or liver, but not in the gastrointestinal tract [23], while in tamoxifen inducible knockout mice THOC5 was also depleted in the gastrointestinal tract.

### THOC5 inactivation results in rapid death

**Figure 2:**
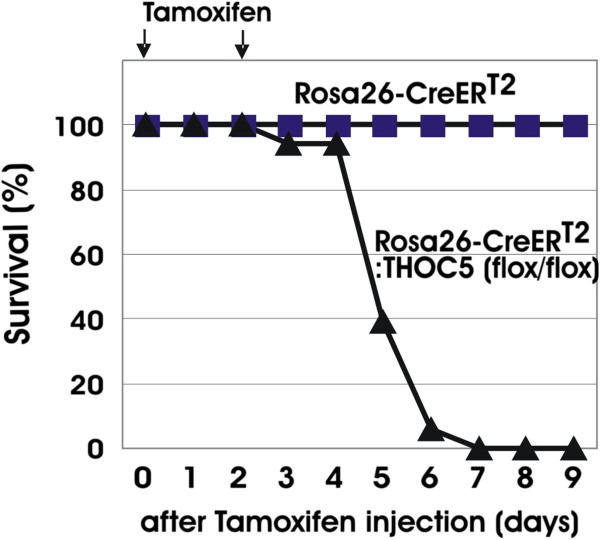
**Deletion of THOC5 is lethal in adult mice.** Rosa26-*Cre*ER^T2^:THOC5(flox/flox) (n = 18) and control Rosa26-*Cre*ER^T2^ (n = 10) mice were injected with tamoxifen. Injection was performed i. p. 2 times at 2-day intervals (arrows: tamoxifen injection).

To study the effects of THOC5 deletion, tamoxifen was injected twice at 2 days intervals (Figure 2, arrows) in 6-week old Rosa26-*Cre*ER^T2^:THOC5 (flox/flox) (n = 18) and Rosa26-*Cre*ER^T2^ (n = 10) control mice. All Rosa26-*Cre*ER^T2^:THOC5 (flox/flox) mice died within 1 week, while control mice did not show any symptoms (Figure 2). Considering the kinetics of THOC5 inactivation after tamoxifen injection and our previous observations in interferon-inducible THOC5 inactivation, we speculated that loss of THOC5 in kidney, liver, bone marrow and/or gut might combine to cause lethality.

### Depletion of the THOC5 gene causes loss of nucleated bone marrow cells

**Figure 3:**
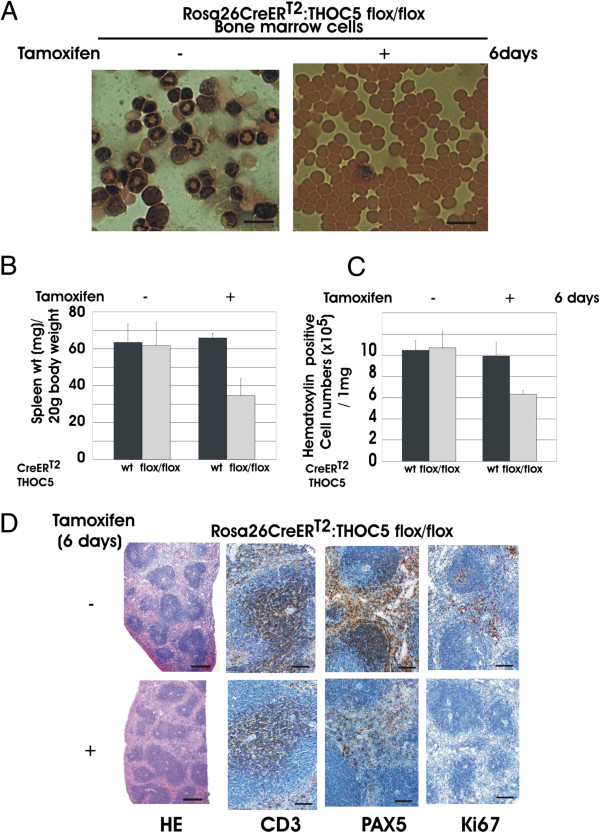
**Depression of the THOC5 gene causes apoptosis of bone marrow cells and reduction of the B cell population in spleen.** Rosa26-*Cre*ER^T2^:THOC5 (flox/flox) (n = 17) and control Rosa26-*Cre*ER^T2^ (n = 14) mice were injected with or without tamoxifen. Injection was performed i. p. 2 times at 2-day intervals. **(A)** Bone marrow cells were spun down onto glass slides and were then stained with May-Grünwald and Haematoxylin (May-Grünwald). We show one example of representative data. Bars represent 10 μm. **(B, C)** Spleen weight was shown as mg/20 g body weight **(B)**, haematoxylin positive cells were counted from 1 mg spleen **(C)**. Mean values from mice treated with tamoxifen: Rosa26-*Cre*ER^T2^:THOC5 (flox/flox) (n = 10) and control Rosa26-*Cre*ER^T2^ (n = 8) or without tamoxifen Rosa26-*Cre*ER^T2^:THOC5 (flox/flox) (n = 7) and control Rosa26-*Cre*ER^T2^ (n = 6) are shown. The error bars represent standard deviation. **(D)** Rosa26-*Cre*ER^T2^:THOC5 (flox/flox) mice were treated with (+) or without (−) tamoxifen and six days after the first tamoxifen injection mouse spleens were fixed in formalin. Paraffin sections were stained by Haematoxylin and eosin (HE) or were supplied for immunohistochemical staining using CD3, PAX5 and Ki67 specific antibodies. Bars represent 800 μm (HE) or 40 μm (immunohistochemical staining).

We have previously shown that using an interferon inducible knockout mouse system THOC5 is essential to sustain hematopoiesis in adult mice. We therefore examined whether tamoxifen inducible THOC5 knockout mice show a similar phenotype. Spleen and bone marrow were isolated from 6- to 9-week old Rosa26-*Cre*ER^T2^:THOC5 (flox/flox) (n = 17) and Rosa26-*Cre*ER^T2^ control (n = 14) mice on day 0 (no tamoxifen) to day 6 after tamoxifen treatment. In agreement with data using interferon inducible THOC5 knockout mice, nucleated bone marrow cells dropped in numbers already 2 days after tamoxifen treatment and after 6 days nucleated cells were very scarce (Figure 3A). Bone marrow cells from control mice did not show any alteration after tamoxifen treatment (data not shown).

### Loss of proliferative cells in THOC5 depleted spleen

We then examined the effect on the spleen. Spleen weight that is normalized to total body weight of control Rosa26-*Cre*ER^T2^ mice was not altered after tamoxifen treatment, while spleen weight of Rosa26-*Cre*ER^T2^:THOC5 (flox/flox) was decreased to approximately half 5 or 6 days after tamoxifen treatment (Figure 3B). It should be noted that at this period, THOC5 depleted mice also lost approximately 20% body-weight. We then counted cell numbers per mg spleen tissue. Six days after tamoxifen treatment white cell numbers were decreased to 60% in THOC5 depleted mice (Figure 3C). To determine which cell type(s) were decreased in spleen by deletion of THOC5, we next examined the morphological alterations to the spleen.

Although no obvious differences in the morphology of the splenic parenchyma were detected between THOC5 depleted and control spleens, there is a clear reduction in the number of B cells visualized by paired box 5 (PAX5) specific staining (Figure 3D). In addition, there is a clear reduction in the fraction of Ki67 positive proliferating cells (Figure 3D). The Ki67 positive cells in the control spleens are mainly in the area in which there is a lack of B-cells, including germinal centers, in the THOC5 depleted spleen. This suggests that THOC5 depleted spleens do not contain proliferating B-cells. On the other hand, no obvious difference was observed in CD3 positive cells before or after tamoxifen treatment (Figure 3D).

### Depletion of the THOC5 gene did not cause pathological alterations in liver or kidney

**Figure 4:**
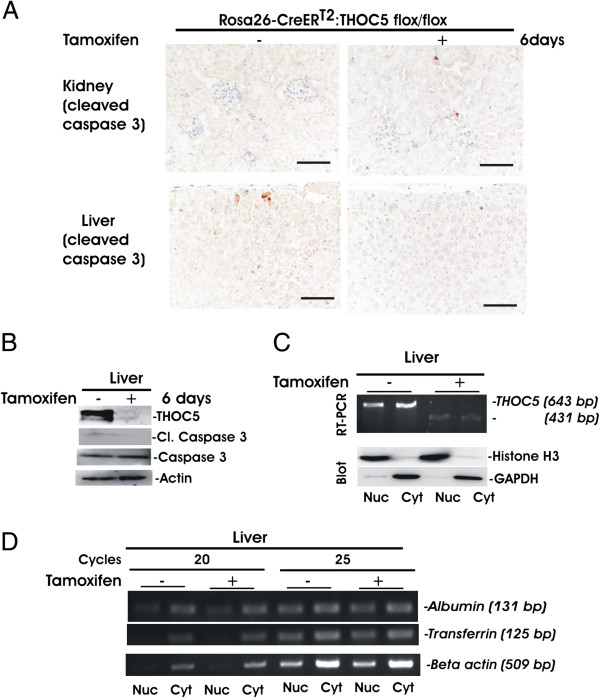
**Depletion of the THOC5 gene did not cause pathological alteration to liver or kidney. (A)** Rosa26-*Cre*ER^T2^:THOC5 (flox/flox) mice were treated with (+) or without (−) tamoxifen and six days after the first tamoxifen injection, kidney and liver were fixed in formalin. Paraffin sections were supplied for immunohistochemical staining using cleaved caspase 3 antibody. Bars represent 100 μm. **(B)** Protein extracts from liver obtained from the same mice as in (A) were supplied for THOC5, cleaved (cl.) caspase 3, caspase 3 and actin specific immunoblot. **(C)** Nuclear (Nuc) or cytoplasmic (Cyt) RNA from 20 μg of liver tissues before or after the tamoxifen treatment were supplied for the *THOC5* specific RT-PCR or nuclear (Nuc) or cytoplasmic (Cyt) protein extracts from same tissues (200 μg of liver tissue) were supplied for GAPDH and Histone H3 specific immunoblot (Blot). **(D)** Aliquots of the same RNAs in **(C)** were supplied for *Albumin, Transferrin* and *beta actin* specific semi-quantitative RT-PCR. Cycles: Number of amplification cycles.

We next examined other organs, kidney and liver. In agreement with our previous data [23], we did not see any pathological alterations, such as inflammation, or any activated caspase 3 positive cells in kidney or liver (Figure 4A and B). Since hepatocytes produce a number of key molecules and enzymes such as albumin or transferrin for the maintenance of life, we examined whether *albumin* or *transferrin* mRNAs were exported into the cytoplasm in the THOC5 depleted liver. We isolated cytoplasmic and nuclear RNAs from 20 μg of liver tissue before and after THOC5 knockdown (Figure 4C,D), and then analyzed *albumin, transferrin* and *actin* expression by semi-quantitative RT-PCR. As control for fractionation, protein extracts of both fractions obtained from 200 μg liver tissues were supplied for Histone H3 (nuclear fraction), and GAPDH (cytoplasmic fraction) specific immunoblot (Figure 4C). In this experiment only spliced forms of mRNA can be detected since we have chosen the primer pair for RT-PCR which is located at two different exons (Table 1). As shown in Figure 4D, upon depletion of THOC5 the export of *albumin* as well as *transferrin* mRNAs were not altered.

**Table 1:** RT-PCR primer pair sequences for selected genes

**Gene**	**Acession number**	**Forward primer**	**Reverse primer**	**RT-PCR (spliced)**	**RT-PCR (unspliced)**
**Alb (exon2-exon3)**	**NM_009654.3**	CAAGAGTGAGATCGCCCATCG	TTACTTCCTGCACTAATTTGGCA	**x**	
**Ascl2(exon1-exon2)**	**NM_008554.3**	AAGCACACCTTGACTGGTACG	AAGTGGACGTTTGCACCTTCA	**x**	
**Birc5 (exon3-exon 4)**	**NM_009689.2**	AGCATAGAAAGCACTCCCCTG	CCACTGTCTCCTTCTCTAAGATCC	**x**	
**Fn1 (exon41-exon43)**	**NM_001276408.1**	TTCAAGTGTGATCCCCATGAAG	CAGGTCTACGGCAGTTGTCA	**x**	
**GAPDH (exon2-exon3)**	**NM_008084.2**	AGGTCGGTGTGAACGGATTTG	TGTAGACCATGTAGTTGAGGTCA	**x**	
**GusB (exon1-exon2)**	**NM_010368.1**	CCGACCTCTCGAACAACCG	GCTTCCCGTTCATACCACACC	**x**	
**Mmp2 (exon1-exon2)**	**NM_008610.2**	CAAGTTCCCCGGCGATGTC	TTCTGGTCAAGGTCACCTGTC	**x**	
**Ptgs2 (exon8-exon9)**	**NM_011198.3**	TTCAACACACTCTATCACTGGC	AGAAGCGTTTGCGGTACTCAT	**x**	
**Snai1 (exon2-exon3)**	**NM_011427.2**	CAAGGAGTACCTCAGCCTGG	GGTCAGCAAAAGCACGGTT	**x**	
**Sox9 (exon2-exon3)**	**NM_011448.4**	CGGAACAGACTCACATCTCTCC	GCTTGCACGTCGGTTTTGG	**x**	
**Sox9 (exon2-intron2)**	**NC_000077.6**	AGTCGGTGAAGAACGGACAAG	GAATGCAAAGTCAGATACTTCTG		**x**
**Beta-actin (exon4-exon5)**	**NM_007393.3**	AACACCCCAGCCATGTACGTAG	GTGTTGGCATAGAGGTCTTTACGG	**x**	
**Beta-actin (exon4-Intron4)**	**AC_000027.1**	GCTGTGCTATGTTGCTCTAGACTT	TTTAGATGGAGAAAGGACTAGGC		**x**
**THOC5 (exon1- exon7)**	**NM_172438.3**	TCTGCCTTTTCACCTGGAAG	CTCGGTACTTTTCTGCCAGC	**x**	
**Trf (exon1-exon2)**	**NM_133977.2**	ACACACACACCGAGAGGAT	GGTATTCTCGTGCTCTGACAC	**x**	
**Wnt11 (exon6-exon7)**	**NM_009519.2**	GGTGGTACACCGGCCTATG	TCACTGCCGTTGGAAGTCTTG	**x**	

**Alb (exon2-exon3):** albumin (131nt); **Ascl2 (exon1-exon2):** Achaete scute complex homolog 2 (115nt); ; **Birc5 (exon3-exon4):** Baculoviral IAP repeat-containing 5 (311nt); **Fn1 (exon41-exon43):** fibronectin1 (154nt); **GAPDH (exon2-exon3):** glyceraldehyde-3 phosphate dehydrogenase (123nt); **GusB (exon1-exon2):** glucouronidase beta (169nt); **Mmp2 (exon1-exon2):** matrix metallopeptidase 2 (171nt); **Ptgs2 (exon8-exon9):** prostaglandin-endoperoxidase synthase 2 (271nt); **Snai1 (exon2-exon3)**: snail homolog 1 (179nt); **Sox9 (exon2-exon3):** SRY-box containing gene 9 (163nt); **Sox9 (exon2-intron2)** (235nt); **beta-actin (exon4-exon5):** (509nt); **beta-actin (exon4-intron4)** (191nt); **THOC5 (exon1-exon7):** THO complex subunit 5 homolog (643nt); **Trf (exon1-exon2):** transferrin (125nt); **Wnt11 (exon6-exon7):** wingless-related MMTV integration site 11 (183nt); (): product size.

In addition, we examined the level of serum albumin before and after tamoxifen treatment. The level of serum albumin was not altered by depletion of THOC5 for 7 days (data not shown). Interestingly, liver expresses THOC5 at relatively high levels on the protein and mRNA levels [9], however our data revealed that THOC5 is not required for maintenance of central liver functions. This may be due to the strong regenerative potency of hepatocytes. We are currently examining the role of THOC5 in regenerating liver. Furthermore, no pathological alterations were found in other organs such as brain, heart, lung, or testicles and no clear inflammatory response was detected in THOC5 depleted organs at any time (data not shown).

### Depletion of the THOC5 gene in gastrointestinal epithelial cells causes severe degeneration

**Figure 5:**
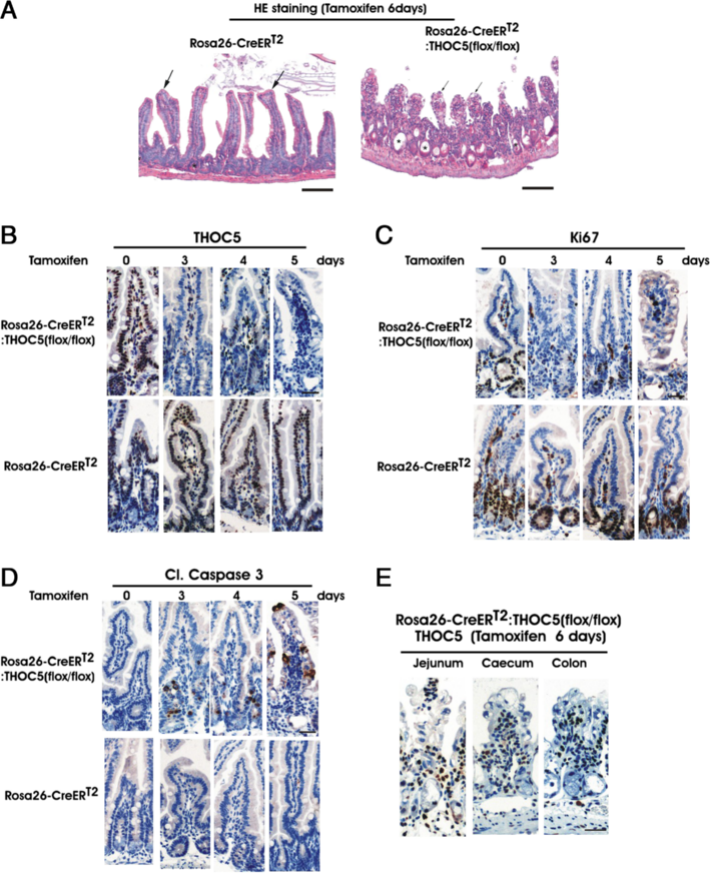
**Depletion of the THOC5 gene causes severe pathological alteration in the gastrointestinal tract.** Rosa26-*Cre*ER^T2^ and Rosa26-*Cre*ER^T2^:THOC5 (flox/flox) mice were treated with (+) or without (−) tamoxifen and jejunum **(A-E),** caecum **(E)**, and colon **(E)** were fixed in formalin. Paraffin sections were stained by hematoxylin and eosin (HE) (A) or were supplied for immunohistochemical staining using THOC5 **(B, E)**, Ki67 **(C)**, and cleaved caspase 3 (Cl. Caspase 3) **(D)** specific antibodies as indicated. Bars represent 400 μm (HE) or 20 μm (immunohistochemical staining).

We next examined the gastrointestinal tract. Gastrointestinal tissues were isolated from 6- to 9-week old Rosa26-*Cre*ER^T2^ and Rosa26-*Cre*ER^T2^:THOC5 (flox/flox) mice on day 0 (no tamoxifen) to day 6 after tamoxifen treatment. Notably, 6 days after tamoxifen treatment the length of small intestine from Rosa26-*Cre*ER^T2^:THOC5 (flox/flox) mice was reduced to half (from 32–33 cm to 17–18 cm), and the length of colon was slightly reduced (by approximately 20%). Depletion of THOC5 caused severe degenerative lesions in the small intestine (Figure 5A). The epithelial villi (Figure 5A, arrows) were disordered and shortened, and the crypts (area of the intestinal stem cells) in THOC5 depleted mice show severe dilatation and necrosis (Figure 5A, asterisk), and sometimes sloughing of cells (crypt abscesses). It should be noted that the changes are minimally associated with influx of inflammatory cells. We then examined THOC5 expression in intestinal epithelial cells. THOC5 is expressed in the nucleus of most intestinal epithelial cells, including the most prevalent enterocytes, in control mice and in non-treated Rosa26-*Cre*ER^T2^:THOC5 (flox/flox) mice, however 3 days after tamoxifen treatment, THOC5 was drastically reduced in the intestinal epithelial cells from Rosa26-*Cre*ER^T2^:THOC5 (flox/flox) mice (Figure 5B). We next stained serial sections with Ki67 and cleaved caspase 3 specific antibodies. Upon depletion of THOC5, Ki67 positive cells were drastically reduced (Figure 5C), while cleaved caspase 3 positive cells were detected in the crypt lesion 3 days after tamoxifen treatment, and 5 days after tamoxifen treatment cleaved caspase 3 positive cells were distributed throughout whole villi (Figure 5D), suggesting that the structural breakdown of stem cell niche, and/or bacterial infection causes apoptosis of more differentiated epithelial cells.

THOC5 expression was drastically reduced not only in small intestine, but also in caecum and colon from THOC5 depleted mice 5 days after tamoxifen treatment (Figure 5E). Furthermore, depletion of THOC5 caused severe degenerative lesions in the small intestine, and some parts of caecum, and to a lesser extent in the large intestine (Figure 5E). Severe pathological alterations in small intestinal epithelial cells may be due to the rapid turnover of epithelial cells, however we observed pathological alterations clearly in caecum and colon, where the rate of turnover and proliferation is also quite high, suggesting that THOC5 is required generally for rapidly proliferating cells in the gut. This is quite different from THOC1 (another member of the THO complex) knockout phenotype which showed that pathological alteration was observed only in stem cells from the small intestine [24]. It is not clear whether the function of THOC1 and/or the efficiency of the tamoxifen inducible system are different than those of THOC5. We did not observe any pathological alterations in tamoxifen treated control mice. The damage of epithelial cells caused translocation of bacteria into the underlying blood vessels (data not shown), which may cause Toll-like receptor activation [25].

### Depletion of the THOC5 gene causes bacterial infection that originates from the gastrointestinal tract

We next isolated blood from heart and incubated it on blood agar plates. No bacterial colonies grew until 2 days after tamoxifen treatment, however colonies (39 and 41 colonies/100 μl blood) were detected in blood from heart in 2 out of 3 mice 4 days after tamoxifen treatment. Five to seven days after tamoxifen treatment, more than 400 bacterial colonies were isolated from 100 μl blood from heart of all THOC5 depleted mice (Table 2). Bacterial colonies were not observed from control mice at any time point. To confirm that the bacteria which were obtained from heart blood originated from the intestines, we next identified bacterial strains in blood from the heart by DNA sequencing using a SOLiD 5500XL system. Ninety-seven% of isolated bacteria were identified as *Gammaproteobacteria* that include *Escherichia coli* that in our mouse colony represents a minor but readily detectable constituent of the gut microbiota (Table 3)*.* This indicates that bacteria detectable in blood indeed originated from the gastrointestinal tract. These data imply that the mice died of sepsis. Since mice also exhibit several defects in immune system (Figure 3), the immune defects may also contribute to the onset of sepsis.

**Table 2:** Depletion of THOC5 gene leads to sepsis

**Rosa26- *Cre *** **ER**^**T2**^**:THOC5 (flox/flox)**	**Number of bacteria colonies (100 μl of blood from heart)**			
	**Day 0**	**Day 2**	**Day 4**	**Days 5–7**
**1**	**0**	**0**	**41**	**>500**
**2**	**0**	**0**	**39**	**420**
**3**	**0**	**0**	**0**	**>500**

Rosa26-*Cre*ER^T2^:THOC5 (flox/flox) (n = 12) mice were injected with tamoxifen. Blood samples were taken from the heart on 0, 2, 4, or 5-7 days after the first injection. Blood samples of 100 μl were plated onto blood agar and colony numbers of bacteria from each mouse are shown.

**Table 3:** Depletion of THOC5 gene causes bacterial infection that originated from the gastrointestinal tract

**Bacteria**	**Percent**
***Gammaproteobacteria***	**97.9**
*Escherichia coli*	76.7
*Proteus (genomic island)*	0.6
*Citrobacter rodentium*	0.25
*Enterobacter spec.*	0.1
Others	20.25

Bacteria derived from mice treated with tamoxifen for 6 days were grown overnight on blood agar and the bacterial DNA was isolated. Bacterial strains were identified by sequencing as detailed in Methods. Percent of bacterial strains from all bacteria are shown.

### THOC5 is indispensable for processing of inducible mRNAs by Wnt or Toll-like receptor signaling

**Figure 6:**
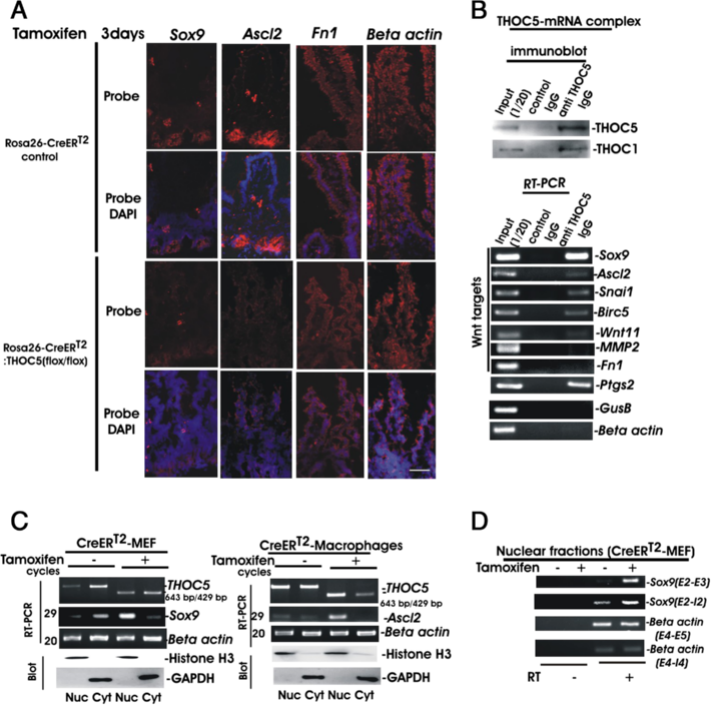
**Wnt signaling downstream genes are THOC5 target mRNAs. (A)***In situ* hybridization: Frozen sections of small intestine from Rosa26-*Cre*ER^T2^:THOC5 (flox/flox) or Rosa26-*Cre*ER^T2^ mice 3 days after tamoxifen treatment were hybridized with *Sox9, Ascl2, Fn1* or *beta*-*actin* specific probes and counterstained with DAPI. Bars represent 40 μm. **(B)** Endogenous THOC5 was immunoprecipitated from the nuclear fraction of mouse embryonic fibroblasts or mouse hepatocellular carcinoma cell line HEPA1-6 using THOC5 specific monoclonal antibody (anti THOC5 IgG) and control mouse IgG (control IgG). THOC5-mRNA complex was analyzed by THOC5 or THOC1 specific immunoblot or bound RNAs were examined by *Sox9, Ascl2, Snai1, Birc5*, *Wnt11*, *MMP2, Fn1, Ptgs2, GusB,* and *beta*-*actin* specific RT-PCR. **(C)** MEF or Bone marrow derived macrophages were isolated from Rosa26-*Cre*ER^T2^:THOC5 (flox/flox) mice. Cells were incubated with (+) and without (−) tamoxifen (10 μM) for three days, and nuclear (Nuc) and cytoplasmic (Cyt) RNAs were then isolated and applied to spliced *Ascl2, Sox9, THOC5* or *actin* specific semi-quantitative RT-PCR using primers as described in Table 1. cDNA from all samples were standardized by adjusting equal levels of *actin* mRNA in both fractions. Protein extracts were supplied for GAPDH and Histone H3 specific immunoblot (Blot). We performed 3 independent experiments and we show one example of representative data. **(D)** Aliquots of nuclear RNAs in **(C)** were supplied for spliced (E2-E3: Exon2-Exon3) or unspliced(E2-I2: Exon2-Intron2) *Sox9,* or spliced (E4-E5: Exon4-Exon5) and unspliced (E4-I5: Exon4-Intron4) *beta actin* specific RT-PCR (primers: Table 1). RT: reverse transcriptase reaction.

Wnt/beta-catenin signaling is a major regulator of homeostatic self-renewal within the intestinal crypt [26,27]. Evidences have accumulated that the THO complex plays an important role in processing of a subset of genes induced by growth factors/cytokines, serum or heat-shock. Thereby, the THO complex is recruited to its target genes and then binds to the target mRNAs [12,16,19,28,29]. We next examined whether Wnt target mRNAs are processed in a THOC5 dependent manner. It must be noted that we have previously identified THOC5 target mRNAs using the mouse embryonic fibroblast system [16]. One of these target mRNAs is SRY-box containing gene 9 *(Sox9)* mRNA which is one of the known Wnt targets [30-35]. We performed *Sox9* specific *in situ* hybridization in THOC5 depleted intestines. *Sox9* transcripts were detected in the intestinal crypt in control intestines, however upon depletion of THOC5, the *Sox9* transcript was reduced (Figure 6A). We further examined other Wnt target mRNAs, achaete-scute complex homolog 2 (*Ascl2)*[31]*,* and fibronectin 1 (*Fn1*) [35]*.* In control intestines Ascl2 is expressed in the intestinal crypt [31], while *Fn1* was detected also in villi (Figure 6A). Upon depletion of THOC5, the *Ascl2* but not *Fn1* transcripts were reduced (Figure 6A). *Beta-actin* transcripts were detected at unaltered levels in control and THOC5 depleted small intestine. These data suggest that some of Wnt target mRNAs may be THOC5 dependent. However, THOC5 depletion partially causes the loss of cellularity in the small intestine, and we therefore cannot rule out that depletion of Wnt target mRNAs is simply due to the pathological alteration of the small intestine or due to a reduction in the numbers of cells expressing these mRNAs. We then further confirmed that Wnt target mRNAs are indeed THO complex dependent by isolating THOC5-mRNAs complex [12,16]. For this experiment, endogenous THOC5 was immunoprecipitated by THOC5 specific monoclonal antibody from the nuclear fraction from mouse embryonic fibroblasts, or mouse hepatocellular carcinoma cell line, HEPA1-6. As control, the same fraction was precipitated by normal mouse IgG. Notably, THOC1 was detected in the THOC5 specific immunoprecipitates, but not in the control IgG precipitates (Figure 6B). THOC5-mRNA complex was examined by RT-PCR using specific primers for detection of 7 Wnt target mRNAs [30-35], namely, the *Sox9, Ascl2,* snail family zinc finger 1 *(Snai1),* wingless-related MMTV integration site 11 *(Wnt11)*, Baculoviral IAP repeat-containing 5 (*Birc5* or *Survivin)*, matrix metallopeptidase 2 (*MMP2)* and *Fn1,* and as controls*, GusB* and *beta-actin* transcripts were examined. As shown in Figure 6B, Wnt target mRNA, *Sox9, Ascl2, Snai1, Wnt11,* and *Survivin*, but not *MMP2* or *Fn1*, were detected in the THOC5-mRNAs complex, indicating that a subset of Wnt target mRNAs are indeed THOC5 dependent. Along the same line, we recently found that cytokine induced immediate-early genes, v-ets erythroblastosis virus E26 oncogene homolog 1 (*Ets1)* or *Ets2*, but not early growth response 1 *(Egr1)*, were THOC5 dependent, indicating that not all induced genes were THOC5 dependent [17]. We then examined one of Toll-like receptor target mRNAs, prostaglandin-endoperoxide synthase 2 (*Ptgs2* or *Cox2). Ptgs2* mRNA was also isolated in the THOC5-mRNA complex (Figure 6B), indicating that Toll-like receptor signalling is also impaired by depletion of THOC5. We next examined whether THOC5 plays a role in processing of Wnt target mRNAs, Sox9 or Ascl2. We isolated nuclear and cytoplasmic RNA from THOC5 depleted mouse embryonic fibroblasts (MEF) or bone marrow derived macrophages derived from Rosa26-*Cre*ER^T2^:THOC5 (flox/flox) mice. *Beta actin* mRNA was used as an internal control for equal amounts of cDNA used for each sample (Figure 6C, *actin*). As control for fractionation, aliquots of protein extracts from each sample were supplied for Histone H3 (nuclear fraction), and GAPDH (cytoplasmic fraction) specific immunoblot (Blot). Spliced *Sox9* and *Ascl2* mRNAs were exported in the presence of THOC5, however, depletion of THOC5 drastically reduced the export (Figure 6C). To determine the effect of THOC5 in the target mRNA processing, we next examined the spliced and unspliced forms of Sox9 mRNA in the nuclear fraction before and after deletion of THOC5. The unspliced form of Sox9 mRNA also accumulated in the nucleus upon depletion of THOC5 (Figure 6D), indicating that THOC5 contributes not only to the mRNA export, but also to the efficient processing of target mRNA or the degradation of unspliced mRNAs.

Presently, it is not clear how the THO complex selects its target mRNAs. Recently, Katahira et al. [19] proposed that THOC5 controls an alternative polyadenylation site choice by recruiting cleavage and polyadenylation specific factor 6, (CPSF6, or CFIm68) on target genes. However, 70-75% of human mRNAs contain a potential alternative polyadenylation site [36]. Moreover, *Snai1* (THOC5 dependent gene) does not contain this site. Alternatively, it has been previously shown in the yeast system that yeast homolog of THOC1, Hpr1 is preferentially required for transcription of either long or GC rich DNA sequences [2]. Interestingly, the coding region (not in the 3′-UTR) of *Sox9*, *Ascl2*, *Snai1*, and *Wnt11* (THOC5 dependent genes) contain GC to a great degree (61-67%), while THOC5 independent genes contain 54-55% GC. However, the target selectivity of THOC5 remains to be studied.

It has been previously shown that depletion of the Wnt target gene, *Ascl2* results in a similar phenotype with pathological alterations of small intestinal epithelial cells [37] to the phenotype observed in THOC5 depleted epithelial cells, however THOC5 depleted mice showed more severe alterations of phenotype than Ascl2 −/− mice. This may be due to the fact that THOC5 knockout causes depletion of the pool of Wnt target mRNAs.

These data imply that some population of genes inducible by growth factors/cytokines, Wnt signaling, and probably also Toll-like receptor signaling are THOC5 dependent, thus THOC5 plays a crucial role in proliferation/differentiation processes in regenerative organs. Furthermore, these data also provide evidence that THOC5 may be a useful tool for modification of the differentiation processes, and for cancer therapy.

## Conclusion

THOC5 is indispensable for processing of only a subset of mRNAs, but plays a key role in processing of mRNAs inducible by Wnt signals. Furthermore, THOC5 is dispensable for general mRNA export in terminally differentiated organs, indicating that multiple mRNA export pathways exist. These data imply that THOC5 may be a useful tool for studying intestinal stem cells, for modifying the differentiation processes and for cancer therapy.

## Methods

### Genotyping for Rosa26-CreER^T2^:THOC5 (flox/flox) and Rosa26-CreER^T2^ mice

Generation of THOC5 (flox/flox) mice has been described previously [23]. Genotyping was performed from tail-derived sample DNA extracted with DirectPCR-Tail Lysis Reagent (Peqlab, Erlangen, Germany) by PCR using THOC5 primers flanking the floxed region with 5′-CCCTCGGCCCCTTTTGAG-3′ and 5′-CAGCACTGGAGCGGGAGATGT-3′ [23]. After crossing with Rosa26 ERT2-*Cre* deleter mice (A kind gift from Anton Berns, Netherlands Cancer Institute), mice were genotyped by PCR using primers Cre1 5′-CCGGGCTGCCACGACCAA-3′ and Cre2 5′-GGCGCGGCAACACCATTTT-3′ for *Cre* gene and primers for floxed THOC5 gene. For the determination of exons 4/5 deletion, exon 4/5 specific PCR primers: 5′-CTGTGTGCACTTCATGACTCTAAAGA-3′ and 5′-GAACTCCAGACATTTGGTGATCTCCT-3′, introns 3/5 specific PCR primers: 5′-TGCTGGCATTGAACTGTG-3′ and 5′-CAGCACTGGAGCGGGAGATGT-3′ and exon 20 specific PCR primers: 5′-GCTATGGAGAGTGAAGTCAACGTGT-3′ and 5′-CTAAAAAGCCGCAGGCACATCTT −3′ were used.

### RT-PCR analysis

RNA was isolated from mouse tissue with the High Pure RNA Isolation kit (Roche Diagnosis, Mannheim, Germany) according to the manufacturer’s instructions. To isolate cytoplasmic RNAs from liver we used NE-PER nuclear and cytoplasmic extraction reagents (Pierce, Thermo Scientific, Germany). Reverse transcription was carried out using oligo dT primers and the Omniscript reverse transcriptase kit (Qiagen, Hilden, Germany) following the instructions provided. Primer pairs for each PCR are shown in Table 1. PCRs were set up according to the following profile: an initial denaturation step of 94°C for 3 minutes, 35 cycles of 94°C for 30 seconds, 60°C for 30 seconds, and 72°C for 30 seconds followed by a final extension step at 72°C for 10 minutes. Separation of the DNA fragments was carried out on 2% (w/v) agarose gels, stained with ethidium bromide (2 μg/ml) and photographed under UV light.

### Tamoxifen injection

Tamoxifen (Sigma, München, Germany) was injected i.p. (1 mg/20 g body weight) into 5–9 week-old mice, two times at 2-day intervals. All animal experiments were carried out according to institutional guidelines approved by the Niedersächsisches Landesamt für Verbraucherschutz und Lebensmittelsicherheit. This study was approved under permit number: 33.14-42502-04-12/0762.

### Hematopoiesis assays

Cytospin preparations were generated from bone marrow cells and contrast was enhanced by May-Grünwald and hematoxylin staining.

### Immunohistochemistry and immunofluorescence

Immunohistochemical studies were performed as detailed previously [38]. Monoclonal Rat anti mouse Ki67 and polyclonal rabbit anti CD3 antibodies were purchased from Dakocytomation (Gloslrup, Denmark) and mouse monoclonal antibody against Pax5 was from BD Biosciences (Heidelberg, Germany). The monoclonal antibody against THOC5 was described previously [15].

### Immunoblot procedures

Details of immunoblotting have been described previously [39]. Mouse organs were extracted with lysis buffer containing 10 mM Tris HCl pH 7.6, 50 mM NaF, 1 mM PMSF, 10 mM EDTA, 1%(w/v) Triton-X 100, 8 M Urea and protease inhibitor cocktail (Sigma, München, Germany). Monoclonal antibody against GAPDH, and polyclonal antibody against actin, were purchased from Santa Cruz Biotechnology, Inc. (Santa Cruz, CA), polyclonal antibodies against cleaved caspase 3, caspase 3, and Histone H3, were from Cell Signaling (Cell Signaling Technology, Beverly, MA), a monoclonal antibody against THOC5 was generated as described previously [15]. Corresponding proteins were visualized by incubation with peroxidase conjugated anti-mouse or anti-rabbit immunoglobulin followed by incubation with SuperSignal West FemtoMaximum Sensitivity Substrate (Pierce Protein Biology Products, Rockford, IL, USA). Results were documented on a LAS4000 imaging system (GE Healthcare Bio-Sciences, Uppsala, Sweden). Signal intensity of chemiluminescence was quantified using TINA 2.0 software (Raytest Isotopenmessgeraete GmbH, Straubenhardt, Germany).

### Bacteria DNA- sequencing

One-hundred microliter samples of blood taken from the heart were plated on blood agar and incubated overnight at 37°C. Bacteria were isolated from the plate and genomic DNA from the bacteria was prepared. Sequencing was performed on a SOLiD 5500XL –System (Life technologies). To generate a bead-coupled fragment library for high throughput sequencing, the bacterial DNA was fragmented and processed using kits and standard protocols of Life technologies (Fragment Library Preparation: 5500 Series SOLiD™ Systems User Guide (Part no. 4460960), 5500 SOLiD™ Fragment Library Core Kit (Part no. 4464412) and EZ Bead™ E20 System Consumables (Part no. 4453094)). Subsequent analysis was performed with the program Genometa [40].

### THOC5-mRNA complex isolation

After three times washing, mouse embryonic fibroblasts or HEPA1-6 cells were lysed with lysis buffer (10 mM Tris, 150 mM NaCl, 1 mM PMSF, 0.5% NP40, protease inhibitor cocktail, (Sigma-Aldrich, München, Germany) and RNase inhibitor), and were then frozen and thawed three times. After centrifugation, supernatants were incubated with mouse monoclonal THOC5 antibody or mouse control IgG with protein G sepharose, and immunoprecipitates were then washed three times. All steps were carried out at 4°C. Bound RNAs were employed for RT-PCR analysis [12].

### *in situ* hybridization

Small intestines were removed from control Rosa26-*Cre*ER^T2^ mice and Rosa26-*Cre*ER^T2^:THOC5 (flox/flox) mice 3 days after tamoxifen injection and flushed with 3% FCS/PBS to remove faecal content. The intestines were cut open longitudinally, rolled into compact circle and frozen in Optimal cutting temperature (OCT) blocks in dry ice. Sections of 5–7 μm thickness were prepared and processed for hybridization as described below. Sections were fixed in 4% paraformaldehyde, dehydrated, digested with proteinase K solution (Thermo Scientific), acetylated by 0.25% acetic anhydride, washed in 2X saline-sodium citrate (SSC) buffer, dehydrated and hybridized with *in vitro* transcribed anti-sense *Sox9* (NM_011448.4: Probe: 557–1137 (nucleotide numbers)), *Ascl2* (NM_00855 4.3 Probe: 1125–1538 (nucleotide numbers)), *Fn1* (NM_001276408.1 Probe: 5021–5370 (nucleotide numbers) and *beta actin* (NM_007393.3: Probe 448–956 (nucleotide numbers)) RNAs overnight or 6 hours in moist chamber at 42°C. The hybridization buffer contains 50% formamide, 0.6 M NaCl, 10 mM Tris.HCl, pH 7.5, 2 mM EDTA, 1× Denhardt’s solution, 1 mg/ml yeast tRNA,10% dextran sulphate, and 10 mM DTT. Sections were then washed at 50°C in prewarmed RNA wash solution I (2X SSC, 50% formamide, 0.1% 2-mercaptoethanol), treated with 20 μg/ml Ribonuclease A and further washed with RNA wash solution I and II (0.1X SSC, 1% 2-mercaptoethanol). After dehydration and drying, sections were incubated for 4 hours at RT in anti-Digoxigenin-Rhodamine (1:100, Roche Diagnostics GmbH). After washing several times in Tris-buffered saline, sections were counterstained with 4′,6-diamidino-2-phenylindole (DAPI). For image analysis, sections were permanently mounted in Mowiol solution.

### Ethics statement

Animal experiments were performed according to the German rules and regulations (Tierschutzgesetz), and approved by the ethics committee of Lower Saxony for care and use of laboratory animals LAVES (Niedersächsisches Landesamt für Verbraucherschutz und Lebensmittelsicherheit). Mice were housed in the central animal facility of Hannover Medical School (ZTL) and were maintained as approved by the responsible Veterinary Officer of the City of Hannover. Animal welfare was supervised and approved by the Institutional Animal Welfare Officer (Tierschutzbeauftragter).

## Competing interests

The authors declare that they have no competing interests.

## Authors’ contributions

SS and DDHT carried out the tamoxifen injection, RT-PCR, Immunoblot, in situ hybridization, cytospin preparation, and prepared figures, SKF generated Rosa26ER^T2^: THOC5(flox/flox) mice, supervised the maintenance of mouse colony, and performed genotyping. AK and AH participated in the design of the study, supervised the study and analyses of data. HC participated cryosection and in situ hybridization and OP participated in its design and coordination, and review of the manuscript. PML and LW performed typing of bacteria strains, and RK performed the pathological analyses. TT participated in the design of the study, contributed to the data analysis, and wrote and finalized the manuscript. All authors participated in the discussion and approved the final manuscript.
